# Virulence of Clinical *Candida* Isolates

**DOI:** 10.3390/pathogens10040466

**Published:** 2021-04-12

**Authors:** Martyna Mroczyńska, Anna Brillowska-Dąbrowska

**Affiliations:** Department of Molecular Biotechnology and Microbiology, Gdańsk University of Technology, Narutowicza 11/12, 80-233 Gdańsk, Poland; martyna.mroczynska@pg.edu.pl

**Keywords:** *Candida* virulence, virulence factors, *Galleria mellonella*

## Abstract

The factors enabling *Candida* spp. infections are secretion of hydrolytic enzymes, adherence to surfaces, biofilm formation or morphological transition, and fitness attributes. The aim of this study was to investigate the correlation between known extracellular virulence factors and survival of *Galleria mellonella* larvae infected with clinical *Candida*. The 25 isolates were tested and the activity of proteinases among 24/24, phospholipases among 7/22, esterases among 14/23, hemolysins among 18/24, and biofilm formation ability among 18/25 isolates was confirmed. Pathogenicity investigation using *G. mellonella* larvae as host model demonstrated that *C. albicans* isolates and *C. glabrata* isolate were the most virulent and *C. krusei* isolates were avirulent. *C. parapsilosis* virulence was identified as varied, *C. inconspicua* were moderately virulent, and one *C. palmioleophila* isolate was of low virulence and the remaining isolates of this species were moderately virulent. According to our study, virulence of *Candida* isolates is related to the expression of proteases, hemolysins, and esterases.

## 1. Introduction

**Figure 1:**
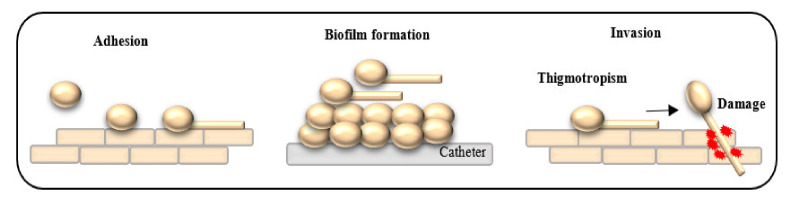
Selected virulence factors of *Candida* spp.

*Candida* spp. are the part of natural microbiota of healthy individuals. However, under conditions of host weakness, *Candida* isolates can become opportunist. The genotyping research of Brillowska-Dąbrowska et al. confirmed that endogenous isolates are the major cause of *Candida* infections [1]. Those infections are the fourth most common hospital-acquired systemic infections in the United States with mortality rates of up to 40% [2]. Progress of medicine paradoxically is a reason of increasing number of opportunistic *Candida* infections. The increasing number of immunocompromised patients, long-term stay at hospital, prolonging patients’ lives, using broad-spectrum antibiotics as prophylaxis and also health-care materials such as catheters and intravenous solutions have contributed to the increase of fungal infections [3]. *Candida* spp. facilitated its invasion and infections by expression of virulence factors (secretion of hydrolytic enzymes, adherence to surfaces, biofilm formation or morphological transition—Figure 1) and fitness attributes [4].

The first crucial step of *Candida* invasion is adherence to host cells. However, adhesion is also important to commensal carriage of these species. Adhesins play a role in facilizing adherence of *Candida* cells to other microorganisms or host cells and abiotic surfaces (medical devices) [5]. The best described adhesins of *C. albicans* are ALS (agglutinin-like sequence) proteins and Hwp1 (hypha-associated glycosylphosphatidylinositol (GPI)-linked) protein. 

*Candida* spp. invade the host cell by invasins presented on the cell surface. These proteins facilitate the host cell to achieve endocytosis by binding to the host ligand. There are known two proteins responsible for invasion: Als3 (plays role of adhesin) and Ssa1 (heat shock protein—Hsp70) [6,7]. Those proteins bind to host E-cadherin and induce endocytosis. However, it has been proven that invasion of *C. albicans* into the host cell relies on active penetration. The molecular mechanism of active penetrations have been undefined yet. It has been established that this process requires viable *C. albicans* hyphae, but it seems that fungal adhesion and physical forces are crucial [8]. However, *C. albicans* hyphae formation mutants also do not express invasins, so it means that both active penetration and endocytosis are depended on hyphae formation [9].

Other virulence factors produced by *Candida* spp. are extracellular enzymes, responsible for tissue adhesion and penetration and thus host invasion. Four different classes of hydrolases (proteases, phospholipases, lipases, and hemolysins) have been so far identified in *Candida* spp. [5].

Proteases degrade different proteins of host cells, such as collagen, keratin, and mucin. These enzymes are also responsible for degradation of antibodies, complements and cytokines [4]. The best characterized proteases are Sap1–Sap10 aspartic proteases. These enzymes can bind to the cell surface or can be secreted to surrounding environment [5]. Aspartic proteases degrade the mucosal membranes of the host tissues and also facilities the degradation of the immunological defense proteins [6]. Moreover, it is considered that increased *Sap* gene expression level is associated with increasing *Candida* virulence and therefore related to clinical symptoms of candidiasis [3].

Phospholipases are responsible for disruption of the cell membrane by hydrolysis of the ester linkages in glycerophospholipids [4]. Four different classes have been so far identified (classes A–D), but only a few members of class B are secreted to the environment [5]. Moreover, the production of phospholipases is strain depending [10,11,12]. 

Hemolysins play a very important role in *Candida* virulence, because they are responsible for destroying red blood cells and iron acquisition. Inorganic elements, such as iron, are essential for the development of *Candida* cells and for the establishment of infection process [3,13]. 

Esterases are enzymes responsible for the hydrolysis of an ester group. However, information about esterases described in the literature are divergent. Esterase production was found among—*C. albicans*, *C. tropicalis*, *C. parapsilosis*, *C. lipolytica*—their low activity was also identified among *C. parapsilosis* [14]. The extracellular lipases play role in lipids digestion for nutrient acquisition, adhesion to host cells and influence on inflammatory process by lysing the competing microflora [3]. In *C. albicans*, 10 different lipases have been found. The research conducted on murine model, showed that inhibition of lipases expression in *C. albicans* and *C. parapsilosis* cells contributed to loss of virulence [15].

Biofilm is the prevalent growth form of microorganisms occurring on catheters, dentures, and mucosal cell surface [16,17]. Biofilm is a community of microorganisms embedded within an extracellular matrix and hyphal cells in the upper part [5]. Biofilm formation ability was identified in *C. auris* [18], *C. albicans, C. glabrata*, *C. parapsilosis*, and *C. tropicalis* [13]. Biofilm is an important virulence factor, responsible to prevent the antimycotic penetration through the matrix and therefore unregular antimycotics penetration into *Candida* cells [19,20], what leads to drug-tolerant and resistant mutants selection [20]. It has been proven that *Candida* biofilm contributes to development of resistance to azoles, polyenes, and pyrimidine analogues [21]. Planktonic cells are more sensitive to antimycotics than the biofilm cells [5]. *Candida* spp. grow budding yeast but they can also grow as pseudohyphae and hyphae [5]. *Candida* polymorphism facilitates host cell invasion due to active penetration by hyphae [17]. *Candida* mutants which are unable to hyphal grow, are less virulent. It was proven that hypha formation is associated with expression of genes encoding virulence factors, such as *HWP1* (hyphal wall protein), *ALS3*, *SAP4*, *SAP5*, etc. [5] 

A model organism for virulence studies of fungal infections should create response of immune system similar to response in human body. In biomedical research the gold standard are mammals (mice, rats, guinea pigs, or rabbits). However, using vertebrates is associated with ethical, logistical and economic considerations. What should be emphasize in virulence study most of the time a huge number of fungal isolates is tested. Those reasons enquired researchers to develop the alternative host models such as embryonated chicken eggs or zebra fish and also invertebrates: *Drosophila melanogaster* [22], *Caenorhabditis elegans* [23], *Blattella germanica* [24], *Bombyx mori* [25]. Recently, *Galleria (G.) mellonella* become used as host models for studying the molecular basis of virulence. 

*G. mellonella* larvae are easy to house and breed on honeycomb or on culture media. The life cycle of *G. mellonella* lasts 6 weeks in optimal conditions. Moreover, the female moth is able to deposits about 1500 eggs [26]. The most important advantage, which is useful for virulence study is that larvae can be incubated in wide range of temperature up to 37 °C. The larvae are small (length approx. 2 cm) so it easy to keep them in laboratory, and the size is appropriate to perform precise application of defined amount of pathogens [27]. The experiments with *G. mellonella* allow to obtain the results during one or two weeks. Finally, *G. mellonella* larvae have ability to synthesize and secrete immune peptides, what allows to use the larvae as a host model [28]. Moreover, low price and ease in maintaining, lack of expensive equipment requirements and finally lack of requirement of ethical approval make *G. mellonella* a new alternative to mammalian models. However, this model has some disadvantages. The lack of defined *G. mellonella* lines and different quality of larvae deepens on distributors contributes to the need of performance detailed controls [29]. The different inoculation volume (10–40 µL) and concentration of pathogens (4 × 10^4^–2 × 10^7^), as well as different protocols and conditions of larvae maintenance make the data difficult to compare between experiments and laboratories. Nevertheless, the larvae of *G. mellonella* have been widely used as virulence models to examine fungal pathogens (*Candida* [30], *Aspergillus* [31], *Histoplasma* [32], *Paracoccidioides* [33], *Fusarium* [34] and *Cryptococcus* [35]) and to evaluate the efficacy of antimicrobial drugs or studies toxins [32,36]. *G. mellonella* larvae were also used to study *C. albicans* virulence [37,38]. What is very important, it was observed that the results obtained using *G. mellonella* are compatible with other model organisms, such as the murine model [31]. 

According to many research *Candida* virulence may vary depending on the species, geographical origin, host reaction and also the stage of infections. The aim of this study was to investigate the correlation between selected, extracellular virulence factors and survival of *G. mellonella* larvae infected with *Candida* isolates.

## 2. Results

### 2.1. Candida Virulence Investigation Using G. mellonella as Host Model

The presented research are based on the group of 25 *Candida* isolates (15 *C. albicans*, 1 *C. glabrata*, 1 *C. inconspicua*, 1 *C. krusei*, 3 *C. palmioleophila*, and 4 *C. parapsilosis*). Among tested isolates, *C. albicans* were the most virulent isolates. According to Kaplan–Meier analysis, 10 *C. albicans* isolates and 1 *C. glabrata* were highly virulent (total 11/25). The remaining 6 *C. albicans* isolates were moderately virulent. *C. parapsilosis* virulence was low, but one *C. parapsilosis* isolates (No. 441) was moderately virulent. We identified only three avirulent isolates: one *C. krusei* isolate and two out of four *C. parapsilosis*. *C. palmioleophila* isolates seemed to have moderate virulence, while *C. inconspicua* was also moderately virulent. Statistically significant differences were observed between the survival among isolates from each groups (*p* value < 0.05). 

**Figure 2:**
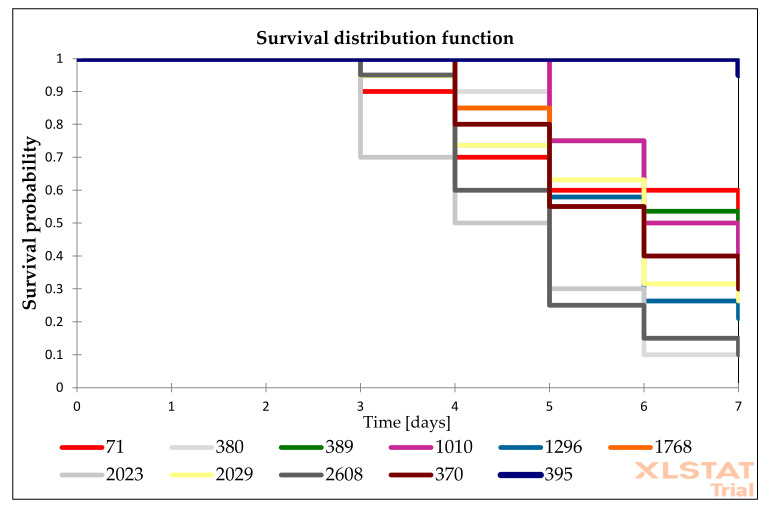
Survival distribution function of *G. mellonella* infected with *Candida* isolates characterized by anidulafungin MIC values of ≤ 0.016 mg/L. The information on which number presented on figure represents what species—is described in Table 1.

**Figure 3:**
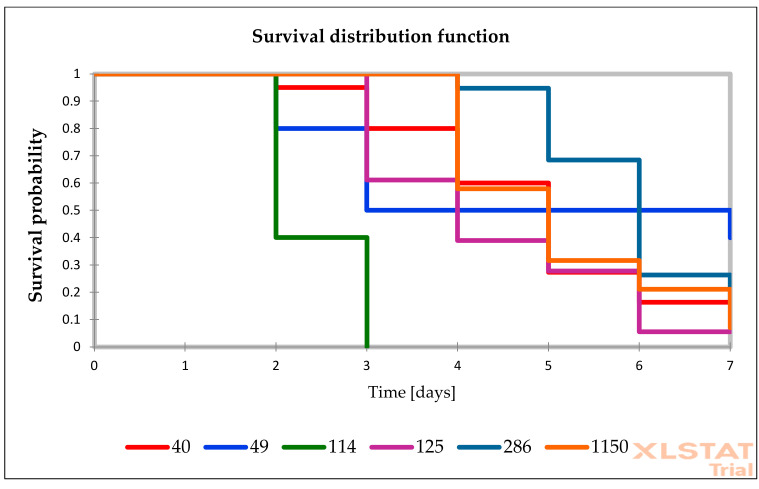
Survival distribution of *G. mellonella* infected with *Candida* isolates characterized by anidulafungin MIC values between 0.031–0.25 mg/L. The information on which number presented on figure represents what species—is described in Table 1.

**Figure 4:**
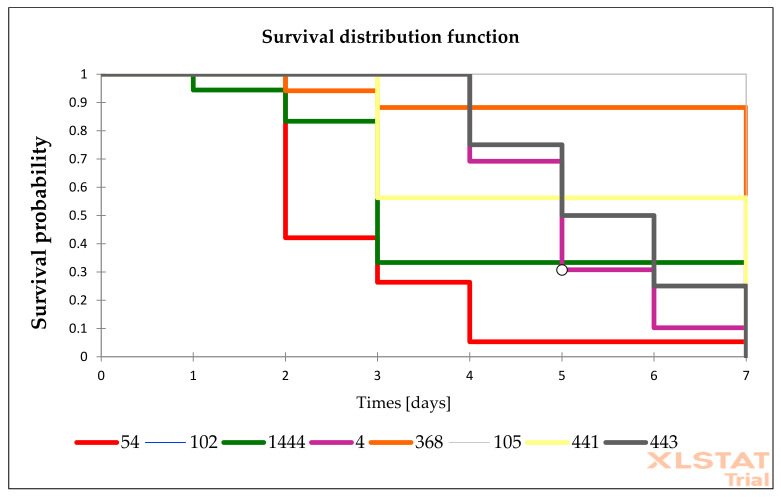
Survival distribution of *G. mellonella* infected with *Candida* isolates characterized by anidulafungin MIC values ≥ 0.5 mg/L. Information regarding number presented in the figure represents what species is described in Table 1.

The survival distribution function of *G. mellonella* larvae after infections of *Candida* isolates is presented in Figure 2, Figure 3 and Figure 4. The survival of larvae after the second, fourth, and sixth day of infections and the standard deviation (SD) from two independent experiment were present in Table S1 (Supplementary Materials). The results of all performed controls are present in Table S1 (Supplementary Materials), while the control results are omitted in the Figures to simplify the picture.

The comparison of the virulence with echinocandin sensitivity suggested that *C. albicans* isolates characterized by the higher MIC values are the most virulent. Nevertheless, echinocandin sensitive *C. albicans* isolates in most cases were moderately or highly virulent. None of *C. albicans* isolates were avirulent. The non-*albicans* isolates with the high MIC values were most often of low virulence. 

### 2.2. Examination of Virulence Factor Production

In Table 1 the comparison of enzymatic activity results and biofilm production with the results of larval survivals are presented according to the MIC values. The Person’s correlation of tested virulence factors are presented in Table S2 in the Supplementary Materials. The enzymatic activity results with their standard deviation are listed in Table S3 in the Supplementary Materials. The exemplary picture of the isolates growing on the specific medium for enzymatic activity testing are presented in Table S4.

Among 18 out of 24 (75%) *Candida* isolates the hemolytic activity was observed, two isolates exhibit low weak activity and one did not grow. The majority of isolates with hemolytic activity belong to the species *C. albicans*. Phospholipase activity was observed among 7/22 isolates (32%), four isolates exhibit a strong activity and three exhibit weak activity. Non phospholipases activity was observed among *C. glabrata*, *C. krusei*, *C. inconspicua*, and *C. parapsilosis* isolates. Proteases activity was identified among all tested isolates, with one exception, *C. albicans* isolate 1768 did not grow. Esterase activity was observed among 14/23 (61%) isolates. The majority of isolates producing esterase enzymes were *C. albicans.* Biofilm formation was observed among 18/25 (72% isolates), only 6 isolates had strong biofilm formation ability and 12 isolates had weak ability.

The distribution of virulence factors for the most virulent isolates of *C. albicans* seemed to be variable. *C. inconspicua* and *C. palmioleophila* both produced biofilm and proteases. Isolates with anidulafungin MIC values of ≥0.5 mg/l had less hemolysins and esterase activity.

Correlation analysis results with Person’s coefficient (Table S2 Supplementary Materials) revealed a positive correlation between the survival of larvae after the sixth day and following enzymes activity: esterase activity (r = 0.621, *p* value = 0.0016), hemolytic activity (r = 0.474, *p* value = 0.019), and protease activity (r = 0.505, *p* value = 0.012). Biofilm formation had a positive correlation with MIC values of anidulafungin (r = 0.502, *p* value = 0.0125), caspofungin (r = 0.422; *p* value 0.04), and micafungin (r = 0.504; r = 0.12). Moreover the positive correlation was observed between esterase activity and MIC values of caspofungin and micafungin. 

## 3. Discussion

The virulence factors were identified in different *Candida* spp. such as: *C. albicans* [39], *C. auris* [40], *C. parapsilosis* complex [41], *C. tropicalis* [4], *C. krusei*, and *C. glabrata* [42]. However, the majority of studies were conducted on *C. albicans* isolates, which are considered the most pathogenic species of *Candida* [3]. In the literature, virulence factors among rare *Candida* species are not well-investigated.

Proteinase activity is observed among 70–100% of clinical *C. albicans* isolates [39,43]. The variable numbers of clinical *C. parapsilosis* isolates producing proteinases were reported (37% [44], 87% [41], and 100% [45]). The obtained data are in accordance with previous reports, due to demonstrate the high proteinase production among all tested isolates such as *C. albicans*, *C. parapsilosis*, and also rare species.

Phospholipase activity of *C. albicans* reported in the literature is variable. In one study, phospholipase activity was identified in 100% of *C. albicans* isolates [43] and in another research the production of these enzymes was observed among 29% [46], 48% [47] or 81% [48] of *C. albicans* isolates. Phospholipase activity was not detected within *C. parapsilosis* isolates [43,45]. In the presented study the phospholipase activity was reported among *C. albicans, C. palmioleophila* isolates and the range was the lowest (32%) among tested enzymes. In this research *C. parapsilosis* did not exhibit phospholipase activity what is in line with the literature data [45]. To the best knowledge of the authors’ there are no information about *C. inconspicua* virulence in the literature. However, in this study we reported that *C. inconspicua* isolate is able to produce this enzyme. 

Esterase activity was reported among *C. albicans, C. tropicalis, C. lipolytica, C. inconspicua,* but only *C. parapsilosis* exhibited weak esterase activity [14]. In the other research esterase activity was identified as low as about 37% of *Candida* isolates [46]. On the other hand, esterase production among 87% of *C. albicans*, 47% of *C. parapsilosis* was detected by Pakshir et al. [49] and within 100% of *C. albicans* and *C. tropicalis* isolates by Slifkin et al. [50]. In our research, esterase activity was identified among 61% of tested isolates and the majority of those isolates were *C. albicans* (12/15).

Hemolysins production seems to be very prevalent and was reported in 100% [47] and 82% [46] of *C. albicans*. The low rate of hemolysins activity was identified among *C. parapsilosis* [4]. In this research hemolysins production was observed among 75% of isolates. Only one out of four isolates *C. parapsilosis* isolates and one of three *C. palmioleophila* isolates had hemolysins activity. 

Another virulence factor contributed to development of *Candida* infections is biofilm formation. According to literature biofilm production contributed to 65% of all human infections. Biofilm formation was identified among 86% [47] or 67% [11] of *C. albicans* isolates. Data obtained during this research are in accordance with the literature, as 72% of all tested isolates produced biofilm. The weak biofilm formation ability was observed among *C. palmioleophila*, and the strong among *C. inconspicua*. The correlation between resistance to antifungals and pathogenicity is not well reported so far in the literature. However, *Candida* biofilm formation is considered to facilitate resistance to antifungal agents [3]. The correlation between fluconazole resistance among *C. tropicalis* and virulence factors as biofilm and phospholipase activity was proven [4]. For the other hand among *C. albicans* isolates negative correlation was identified between fluconazole MICs and biofilm formation [51]. In this research the positive correlation between biofilm formation and susceptibility to echinocandin was reported.

Virulence and pathogenicity potential of clinical *Candida* spp. collected from Polish hospital were investigated in vitro using *G. mellonella* larvae as a host model. 

The literature reports on research conducted on *Candida* spp. using *G. mellonella* larvae as a host model is focused mainly on *C. albicans* virulence [52,53,54]. However, this host model was also used for virulence investigation among *C. parapsilosis, C. krusei* [36], *C. glabrata*, and *C. tropicalis* [55]. The virulence of *Candida* spp. tested in *G. mellonella* seems to be species dependent [53]. 

After infections of *G. mellonella* with 10^5^ cells of *C. albicans, C. dubliniensis, C. parapsilosis*, and *C. tropicalis* the larvae mortality was 100% after 4 days of incubation at 37 °C. Lower mortality rates were observed among: *C. lusitaniae* (87%), *C. norvegensis* (37%), *C. krusei* (25%), and *C. glabrata* (20%). A different mortality rates were reported after 3 days of incubation at 30 °C (90% for *C. albicans*, 70% for *C. tropicalis*, 45% for *C. parapsilosis* and only 20% for *C. krusei*) [52]. 

The obtained results showed that data on *G. mellonella* survivals after infections can be employed to differentiate virulence between *Candida* species. The data obtained in this research presented the highest virulence among *C. albicans* isolates and the lowest among *C. krusei* isolates. This results are in line with literature—*C. albicans* was described as the most pathogenic species and *C. krusei* [36] as almost avirulent. According to the best knowledge of the authors this is the first examination report of the virulence of *C. palmioleophila* and *C. inconspicua* in the *G. mellonella* model. We identified *C. inconspicua* as moderately virulent, one *C. palmioleophila* isolate as low, and the remaining isolates of this species as moderately virulent. *C. parapsilosis* virulence was identified as varied, the majority of isolates were avirulent, only one isolate was moderately virulent. According to the literature virulence of the species from psilosis group is different—*C. parapsilosis sensu stricto* was the most virulent and *C. metapsilosis* was the weakest virulent species [56]. 

The correlation between mortality of the infected larvae and production of *Candida* virulence factors are not well investigated. This correlation was proven for biofilm [37,57] and proteases [58]. However, studies of 739 *Candida* isolates did not show the biofilm correlation with *G. mellonella* larval model [54]. Our research indicated that pathogenicity of *Candida* isolates examined in *G. mellonella* larvae is correlated with proteinase, hemolysins, and esterase activity but correlation between biofilm and larvae mortality were not confirmed.

## 4. Materials and Methods

### 4.1. Candida spp.

In presented studies 25 *Candida* isolates (15 *C. albicans*, 1 *C. glabrata*, 1 *C. inconspicua*, 1 *C. krusei*, 3 *C. palmioleophila*, and 4 *C. parapsilosis*) were tested. To exclude the contamination of another species, and cross contamination of *Candida* spp., all samples were cultured on CHROMagar medium (GRASO, Starograd Gdanski, Poland). The isolates that grew on the chromogenic medium in more than one color were regrown on SAB medium several times and the purities of the colony was investigated again. Species identification was carried out based on rRNA fragment sequencing, according to the procedure described by Mroczyńska [59]. 

Tested isolates were divided into three groups depending on the MIC values of anidulafungin to simplify data analysis. Echinocandins MIC values were determined according to European Committee on Antimicrobial Susceptibility Testing recommendation.

### 4.2. Candida spp. Virulence Investigation Using G. mellonella as Host Model

The protocol was based on Fuchs et al. procedure [27]. All tested isolates were grown overnight in 10 mL liquid Sabouraud (SAB) dextrose medium (BTL, Warszawa, Poland). Then 2 mL of inoculum was transferred to Eppendorf tubes and then centrifuged 1 min at 5000× *g*. The pellet of cells, after removed of the supernatant, was twice washed with PBS (Sigma Aldrich, Darmstadt, Germany). Then the pellet was suspended in 1 mL of PBS. Such prepared suspension was used to count the *Candida* cells with hemocytometer. The cell concentration was adjusted to appropriate amount of cell per microliter in sterile PBS. 

Prepared inoculum of *Candida* isolate was injected into individual larva (Owadodajnia, Gdynia, Poland) in the last left proleg with final concentration 5 × 10^5^ yeast cells/larva. For injection 10 µL of inoculum Hamilton syringe (Cat. No. 701RN 10 µL) was used. Before injections, the area of larvae was sterilized by ethanol. Every experiment was repeated twice and 10 larvae was used per one strain in one experiment. During experiments two control groups of 10 larvae were examined. The control experiments were performed to check the correctness of the injection process, the quality and sterility of PBS and to monitor the quality of delivered larvae. Moreover, also the control of injection of larva was performed (larva were only pierced).

After injection the larvae were transferred to a Petri dish containing a Whatman filter to recover (5–10 min). Groups of larvae injected with particular isolates were transferred to new separate Petri dishes. Infected larvae were maintain at 37 °C and scored for viability daily during seven days. Dead larvae and pupa were removed and the death and metamorphosis were noted. The remaining larvae were returned to the incubator. The endpoint of the experiment was when all larvae were death or transited from larva into pupa.

The results were presented as Kaplan–Meier survival plots and evaluated using Mann–Whitney (two-samples Wilcoxon) test. The *p* value < 0.05 was considered as statistically significant. All calculations were performed using XLSTAT computer program (demo version).

### 4.3. Examination of Virulence Factors Production

For determination of virulence factor production the following agar media were prepared. To determine the ability of phospholipase secretion the SAB medium (1.3%) supplemented by 1.17% NaCl (POCH, Gliwice, Poland), 0.011% CaCl_2_ (POCH, Gliwice, Poland), and 2% of the supernatant of egg yolk emulsion (Bio-maxima, Gdansk, Poland) was used. For positive phospholipase activity the appearance of a whitish zone of precipitation around colony was observed [39,60].

For detection of protease activity specific agar medium was prepared: 2% agar (BTL, Poland), 1.17% yeast carbon base (BTL, Warszawa, Poland), 0.01% yeast extract (BTL, Warszawa, Poland), and 0.2% bovine serum albumin (Sigma-Aldrich, Darmstadt, Germany). Yeast carbon base and BSA were sterilized by 0.22 µL filter. The presence of the opaque halo of degradation around the colonies indicate positive protease activity [61].

Hemolysin activity was examined on SAB medium with 3% glucose (A&A Biotechnology, Gdynia, Poland) and 7% sheep blood (pH = 5.6; Bio-maxima, Poland). The sheep blood was added when the autoclaved reagents were cooling to 50 °C. The presence of the translucent zone around colonies indicate the positive hemolytic activity [61,62].

Esterases activity was detected on medium which consist of 10% of peptone (BTL, Warszawa, Poland), 5% of NaCl, 0.1% of CaCl_2_ and 15% of agar with addition of Tween 80 (Pol-Aura, Gdansk, Poland) added after sterilization. The esterase production was determined by halo zones around the colony when observed against the transmitted light [50].

Culture were initiated on the surface of all mediums the 5 μL of *Candida* cell suspension in PBS (Sigma-Aldrich, Darmstadt, Germany) was placed. The density of culture were adjusted to 0.5 McFarland standard—1–5 × 10^6^ CFU/mL yeast cells. The inoculated plates were incubate at 37 °C for 48 h. After incubation, the results were read visually as described above. 

For all virulence factors the diameter of zone was measured and the activity were calculated used formula: P_z_ (precipitation zone): the phospholipase activity value (P_z_) = (colony diameter)/(colony diameter with zone of precipitation). The results were classified as non-activity (P_z_ = 1), weak (0.64 < P_z_ < 0.99) and strong activity (P_z_ ≤ 0.63) [39,61]. The PR_z_ index was determined as protease activity, H_z_ index as hemolytic activity and E_z_ index as esterase activity. Those values were determined and interpreted in the same way as phospholipase activity described above as example.

*Candida* biofilm investigation was based on the procedure described by Silva et al. [17]. Two hundred microliters of *Candida* cell suspension (0.5 McFarland standard—1–5 × 10^6^ CFU/mL yeast cells) in SAB broth medium were transferred to each well of 96-well polystyrene microtiter plates (Medlab-Products, Raszyn, Poland). As negative control in empty wells medium without cells was placed. Next the plates were incubated at 37 °C on a shaker. After 24 h, 50 µL of the fresh SAB broth medium was added to the culture and then incubated for a further 24 h. After incubation the wells were washed twice with sterile water to remove the non-adherent cells. Biofilm was fixed by adding 200 µL of methanol (POCH, Gliwice, Poland) and incubated 15 min. Then the liquid was removed and the plates allowed to dry. Next, 200 µL of crystal violet (0.5% *v/v*; Sigma Aldrich, Darmstadt, Germany) were added to each well and incubated for further 5 min at room temperature. After removing of stain, wells were washed twice with sterile water. To release and dissolve the stain, 200 µl of acetic acid (POCH, Gliwice, Poland) was added into the wells. The absorbance of the solution was read at 600 nm using microtiter plate reader (Eppendorf BioSpectrometer^®^ kinetic, Eppendorf, Hamburg, Germany). The absorbance of negative control were subtracted from all values for tested wells. All experiments were repeated three times and data are presented as the arithmetic mean and the standard deviation of the data. In the experiment the reference strain of *C. albicans* (ATCC 9028) was used, which is biofilm negative [63]. To determine if the isolates produce biofilm the cut off OD were define using the OD of reference strain. The results were classified as

OD ≤2 × OD _reference_ − biofilm negative

2 × OD _reference_ < OD <3 × OD _reference_ − weak biofilm production

OD ≥3 × OD _reference_ − strong biofilm production.

The correlation coefficient (Person’s r) was used to measure the correlation between virulence factors, survival of larvae and also MIC values. A perfect correlation is observed when r = 1. When r = 0 it means that there is no correlation and when r = −1, then the negative correlation is observed.

## 5. Conclusions

The most important conclusion of performed experiments are:

- *C. albicans* isolates were the most virulent and produce the highest number of extracellular virulence factors;
- A positive correlation between the biofilm formation and the MIC values of echinocandins was confirmed among the tested *Candida* isolates;
- The virulence of *Candida* isolates is related to the expression of proteases, hemolysins, and esterases;

## Figures and Tables

**Table 1:** Comparison of enzymatic activity and biofilm formation of *Candida* isolates with the larvae survival after six days of those isolates’ infection. Tested isolates were divided into three groups depending on the MIC values of anidulafungin.

MIC Range	*Candida* spp.	Isolate No.	Hemolytic Activity	Phospholipase Activity	Protease Activity	Esterase Activity	Biofilm	Survival
			H_z_ Value	P_z_ Value	PR_z_ Value	E_z_ Value	Abs_600_	6th Day
MIC value ≤ 0.016 mg/L	*C. albicans*	71	0.59	1.0	0.35	0.40	0.51	0.6
	*C. albicans*	380	0.52	1.0	0.39	0.45	0.89	0.1
	*C. albicans*	389	0.48	1.0	0.36	1.0	1.89	0.54
	*C. albicans*	1010	0.48	0.48	0.39	0.44	1.27	0.50
	*C. albicans*	1296	0.44	0.80	0.48	0.55	0.80	0.26
	*C. albicans*	1768	- *	-	-	-	0.11	0.40
	*C. albicans*	2023	1.0	0.48	0.36	0.44	0.49	0.15
	*C. albicans*	2029	0.57	0.88	0.35	0.37	1.34	0.32
	*C. albicans*	2608	0.48	1.0	0.38	0.46	1.04	0.15
	*C. palmioleophila*	370	0.67	0.51	0.37	1.0	1.15	0.40
	*C. parapsilosis*	395	1.0	1.0	0.50	1.0	0.42	1.0
MIC value 0.31–0.25 mg/L	*C. albicans*	40	0.44	1.0	0.37	0.53	0.97	0.16
	*C. albicans*	49	0.56	1.0	0.34	0.48	0.24	0.40
	*C. albicans*	114	0.67	-	0.45	0.53	0.12	0.0
	*C. albicans*	125	0.61	0.81	0.36	0.39	1.05	0.056
	*C. albicans*	286	0.48	1.0	0.35	0.44	0.97	0.33
	*C. glabrata*	1150	0.50	-	0.35	1.0	0.81	0.21
MIC value ≥ 0.5 mg/L	*C. albicans*	54	0.48	1.0	0.32	-	1.56	0.0
	*C. krusei*	102	0.52	1.0	0.40	1.0	0.98	1.0
	*C. palmioleophila*	4	1.0	0.63	0.40	0.52	1.05	0.44
	*C. palmioleophila*	368	1.0	1.0	0.48	1.0	0.97	0.90
	*C. parapsilosis*	105	1.0	1.0	0.59	1.0	2.33	1.0
	*C. parapsilosis*	441	0.59	1.0	0.39	0.63	1.01	0.7
	*C. parapsilosis*	443	1.0	1.0	0.35	1.0	0.69	0.65
	*C. inconspicua*	1444	0.56	1.0	0.48	1.0	1.58	0.40

* The growth of isolates was not observed or was insufficient. Hz—index of hemolytic activity; Pz—index of phospholipase activity; PRz—index of protease activity; Ez—index of esterase; Abs_600_—absorbance (600 nm); 6th—larvae survival after 6 days of infection. Dark red marks a strong activity (≤0.63), light red marks a weak activity (0.64 < value < 0.99) and green marks a non—activity (value = 1) of extracellular enzymes. Violet marks a strong biofilm production (OD value ≥ 1.14), yellow weak biofilm production (0.76 < OD value < 1.14) and blue marks negative biofilm production (OD value ≤ 0.76). Dark red marks a survival of less than 0.4 (highly virulent), light red marks a survival rate between 0.4–0.7 (moderately virulent). The survival ≥ 0.7 to 0.95 (low virulence) is marked with green. The survival equal to 1 is marked in yellow.

## Data Availability

Data sharing is not applicable to this article.

## Supplementary Materials

The following are available online at https://www.mdpi.com/article/10.3390/pathogens10040466/s1, Table S1. The survival of larvae infected with individual *Candida* isolates after the second, fourth, and sixth day of infections and the standard deviation from two independent experiment; Table S2. The Person’s correlation and *p* value between tested variabilities; Table S3. Enzymatic activity from *Candida* isolates and the standard deviation from independent experiments; Table S4. Exemplary pictures of the virulence factor study on various substrates.

## References

[1] Brillowska-Dabrowska A., Bergmann O., Jensen I.M., Jarløv J.O., Arendrup M.C. (2010). Typing of Candida Isolates from Patients with Invasive Infection and Concomitant Colonization. Scand. J. Infect. Dis..

[2] Pfaller M.A., Diekema D.J. (2007). Epidemiology of Invasive Candidiasis: A Persistent Public Health Problem. Clin. Microbiol. Rev..

[3] Sardi J.C.O., Scorzoni L., Bernardi T., Fusco-Almeida A.M., Mendes Giannini M.J.S. (2013). Candida Species: Current Epidemiology, Pathogenicity, Biofilm Formation, Natural Antifungal Products and New Therapeutic Options. J. Med. Microbiol..

[4] Deorukhkar S.C., Saini S., Mathew S. (2014). Virulence Factors Contributing to Pathogenicity of Candida Tropicalis and Its Antifungal Susceptibility Profile. Int. J. Microbiol..

[5] Mayer F.L., Wilson D., Hube B. (2013). *Candida Albicans* Pathogenicity Mechanisms. Virulence.

[6] Sun J.N., Solis N.V., Phan Q.T., Bajwa J.S., Kashleva H., Thompson A., Liu Y., Dongari-Bagtzoglou A., Edgerton M., Filler S.G. (2010). Host Cell Invasion and Virulence Mediated by Candida Albicans Ssa1. PLoS Pathog..

[7] Yang W., Yan L., Wu C., Zhao X., Tang J. (2014). Fungal Invasion of Epithelial Cells. Microbiol. Res..

[8] Wächtler B., Wilson D., Haedicke K., Dalle F., Hube B. (2011). From Attachment to Damage: Defined Genes of Candida Albicans Mediate Adhesion, Invasion and Damage during Interaction with Oral Epithelial Cells. PLoS ONE.

[9] Swidergall M., Filler S.G. (2017). Oropharyngeal Candidiasis: Fungal Invasion and Epithelial Cell Responses. PLoS Pathog..

[10] Cafarchia C., Romito D., Coccioli C., Camarda A., Otranto D. (2008). Phospholipase Activity of Yeasts from Wild Birds and Possible Implications for Human Disease. Med. Mycol..

[11] Furlaneto-Maia L., Specian A.F., Bizerra F.C., de Oliveira M.T., Furlaneto M.C. (2008). In Vitro Evaluation of Putative Virulence Attributes of Oral Isolates of Candida Spp. Obtained from Elderly Healthy Individuals. Mycopathologia.

[12] Galán-Ladero M.A., Blanco M.T., Sacristán B., Fernández-Calderón M.C., Pérez-Giraldo C., Gómez-García A.C. (2010). Enzymatic Activities of Candida Tropicalis Isolated from Hospitalized Patients. Med. Mycol..

[13] Silva S., Negri M., Henriques M., Oliveira R., Williams D.W., Azeredo J. (2012). Candida Glabrata, Candida Parapsilosis and Candida Tropicalis: Biology, Epidemiology, Pathogenicity and Antifungal Resistance. FEMS Microbiol. Rev..

[14] Kumar C., Menon T., Nalini S., Thirunarayan M.A., Rajasekaran S., Venkatadesikalu M. (2006). Esterase Activity of Candida Species Isolated from Immunocompromised Hosts. Eur. PMC.

[15] Gácser A., Trofa D., Schäfer W., Nosanchuk J.D. (2007). Targeted Gene Deletion in *Candida Parapsilosis* Demonstrates the Role of Secreted Lipase in Virulence. J. Clin. Investig..

[16] Al-Fattani M.A. (2006). Biofilm Matrix of Candida Albicans and Candida Tropicalis: Chemical Composition and Role in Drug Resistance. J. Med. Microbiol..

[17] Silva S., Henriques M., Martins A., Oliveira R., Williams D., Azeredo J. (2009). Biofilms of Non-Candida Albicans Candida Species: Quantification, Structure and Matrix Composition. Med. Mycol..

[18] Borman A.M., Szekely A., Johnson E.M. (2016). Comparative Pathogenicity of United Kingdom Isolates of the Emerging Pathogen Candida Auris and Other Key Pathogenic Candida Species. mSphere.

[19] Fanning S., Mitchell A.P. (2012). Fungal Biofilms. PLoS Pathog..

[20] Mroczyńska M., Brillowska-Dąbrowska A. (2020). Review on Current Status of Echinocandins Use. Antibiotics.

[21] Sanglard D. (2016). Emerging Threats in Antifungal-Resistant Fungal Pathogens. Front. Med..

[22] Stroschein-Stevenson S.L., Foley E., O’Farrell P.H., Johnson A.D. (2005). Identification of Drosophila Gene Products Required for Phagocytosis of Candida Albicans. PLoS Biol..

[23] Ortega-Riveros M., De-la-Pinta I., Marcos-Arias C., Ezpeleta G., Quindós G., Eraso E. (2017). Usefulness of the Non-Conventional Caenorhabditis Elegans Model to Assess Candida Virulence. Mycopathologia.

[24] Nasirian H. (2017). Contamination of Cockroaches (Insecta: Blattaria) to Medically Fungi: A Systematic Review and Meta-Analysis. J. Mycol. Médicale.

[25] Nwibo D.D., Hamamoto H., Matsumoto Y., Kaito C., Sekimizu K. (2015). Current Use of Silkworm Larvae (Bombyx Mori) as an Animal Model in Pharmaco-Medical Research. Drug Discov. Ther..

[26] Jorjão A.L., Oliveira L.D., Scorzoni L., Figueiredo-Godoi L.M.A., Cristina A., Prata M., Jorge A.O.C., Junqueira J.C. (2018). From Moths to Caterpillars: Ideal Conditions for Galleria Mellonella Rearing for in Vivo Microbiological Studies. Virulence.

[27] Fuchs B.B., O’Brien E., Khoury J.B.E., Mylonakis E. (2010). Methods for Using Galleria Mellonella as a Model Host to Study Fungal Pathogenesis. Virulence.

[28] Mowlds P., Kavanagh K. (2008). Effect of Pre-Incubation Temperature on Susceptibility of Galleria Mellonella Larvae to Infection by Candida Albicans. Mycopathologia.

[29] Binder U., Maurer E., Lass-Flörl C. (2016). Galleria Mellonella: An Invertebrate Model to Study Pathogenicity in Correctly Defined Fungal Species. Fungal Biol..

[30] Brennan M., Thomas D.Y., Whiteway M., Kavanagh K. (2002). Correlation between Virulence of Candida Albicans Mutants in Mice and Galleria Mellonella Larvae. FEMS Immunol. Med. Microbiol..

[31] Slater J.L., Gregson L., Denning D.W., Warn P.A. (2011). Pathogenicity of Aspergillus Fumigatus Mutants Assessed in Galleria Mellonella Matches That in Mice. Med. Mycol..

[32] Thomas R.J., Hamblin K.A., Armstrong S.J., Müller C.M., Bokori-Brown M., Goldman S., Atkins H.S., Titball R.W. (2013). Galleria Mellonella as a Model System to Test the Pharmacokinetics and Efficacy of Antibiotics against Burkholderia Pseudomallei. Int. J. Antimicrob. Agents.

[33] Thomaz L., García-Rodas R., Guimarães A.J., Taborda C.P., Zaragoza O., Nosanchuk J.D. (2013). Galleria Mellonella as a Model Host to Study Paracoccidioides Lutzii and Histoplasma Capsulatum. Virulence.

[34] Fallon J.P., Troy N., Kavanagh K. (2011). Pre-Exposure of Galleria Mellonella Larvae to Different Doses of Aspergillus Fumigatus Conidia Causes Differential Activation of Cellular and Humoral Immune Responses. Virulence.

[35] Firacative C., Duan S., Meyer W. (2014). Galleria Mellonella Model Identifies Highly Virulent Strains among All Major Molecular Types of Cryptococcus Gattii. PLoS ONE.

[36] Scorzoni L., de Lucas M.P., Mesa-Arango A.C., Fusco-Almeida A.M., Lozano E., Cuenca-Estrella M., Mendes-Giannini M.J., Zaragoza O. (2013). Antifungal Efficacy during Candida Krusei Infection in Non-Conventional Models Correlates with the Yeast in Vitro Susceptibility Profile. PLoS ONE.

[37] Borghi E., Romagnoli S., Fuchs B.B., Cirasola D., Perdoni F., Tosi D., Braidotti P., Bulfamante G., Morace G., Mylonakis E. (2014). Correlation between Candida Albicans Biofilm Formation and Invasion of the Invertebrate Host Galleria Mellonella. Future Microbiol..

[38] Fuchs B.B., Eby J., Nobile C.J., El Khoury J.B., Mitchell A.P., Mylonakis E. (2010). Role of Filamentation in Galleria Mellonella Killing by Candida Albicans. Microbes Infect..

[39] De Paula Menezes R., de Melo Riceto É.B., Borges A.S., de Brito Röder D.V.D., dos Santos Pedroso R. (2016). Evaluation of Virulence Factors of Candida Albicans Isolated from HIV-Positive Individuals Using HAART. Arch. Oral Biol..

[40] Larkin E., Hager C., Chandra J., Mukherjee P.K., Retuerto M., Salem I., Long L., Isham N., Kovanda L., Borroto-Esoda K. (2017). The Emerging Pathogen Candida Auris: Growth Phenotype, Virulence Factors, Activity of Antifungals, and Effect of SCY-078, a Novel Glucan Synthesis Inhibitor, on Growth Morphology and Biofilm Formation. Antimicrob. Agents Chemother..

[41] Neji S., Hadrich I., Trabelsi H., Abbes S., Cheikhrouhou F., Sellami H., Makni F., Ayadi A. (2017). Virulence Factors, Antifungal Susceptibility and Molecular Mechanisms of Azole Resistance among Candida Parapsilosis Complex Isolates Recovered from Clinical Specimens. J. Biomed. Sci..

[42] Majumdar T., Mullick J., Bir R., Roy J., Sil S. (2016). Determination of Virulence Factors and Biofilm Formation among Isolates of Vulvovaginal Candidiasis. J. Med. Sci..

[43] De Souza Ramos L., Barbedo L.S., Silva L.A.B., dos Santos A.L.S., Pinto M.R., da Graça Sgarbi D.B. (2015). Protease and Phospholipase Activities of “Candida” Spp. Isolated from Cutaneous Candidiasis. Rev. Iberoam. Micol..

[44] Da Silva B.V., Silva L.B., de Oliveira D.B.C., da Silva P.R., Ferreira-Paim K., Andrade-Silva L.E., Silva-Vergara M.L., Andrade A.A. (2015). Species Distribution, Virulence Factors, and Antifungal Susceptibility Among Candida Parapsilosis Complex Isolates Recovered from Clinical Specimens. Mycopathologia.

[45] Abi-Chacra É.A., Souza L.O.P., Cruz L.P., Braga-Silva L.A., Gonçalves D.S., Sodré C.L., Ribeiro M.D., Seabra S.H., Figueiredo-Carvalho M.H.G., Barbedo L.S. (2013). Phenotypical Properties Associated with Virulence from Clinical Isolates Belonging to the Candida Parapsilosis Complex. FEMS Yeast Res..

[46] Kalaiarasan K., Singh R., Chaturvedula L. (2018). Changing Virulence Factors among Vaginal Non-Albicans Candida Species. Indian J. Med. Microbiol..

[47] Udayalaxmi J., Shenoy N. (2016). Comparison Between Biofilm Production, Phospholipase and Haemolytic Activity of Different Species of Candida Isolated from Dental Caries Lesions in Children. J. Clin. Diagn. Res..

[48] Fule S.R., Das D., Fule R.P. (2015). Detection of Phospholipase Activity of Candida Albicans and Non Albicans Isolated from Women of Reproductive Age with Vulvovaginal Candidiasis in Rural Area. Indian J. Med. Microbiol..

[49] Pakshir K., Zomorodian K., Karamitalab M., Jafari M., Taraz H., Ebrahimi H. (2013). Phospholipase, Esterase and Hemolytic Activities of Candida Spp. Isolated from Onychomycosis and Oral Lichen Planus Lesions. J. Mycol. Médicale.

[50] Slifkin M. (2000). Tween 80 Opacity Test Responses of Various Candida Species. J. Clin. Microbiol..

[51] El-Houssaini H.H., Elnabawy O.M., Nasser H.A., Elkhatib W.F. (2019). Correlation between Antifungal Resistance and Virulence Factors in Candida Albicans Recovered from Vaginal Specimens. Microbial. Pathog..

[52] Cotter G., Doyle S., Kavanagh K. (2000). Development of an Insect Model for the in Vivo Pathogenicity Testing of Yeasts. FEMS Immunol. Med. Microbiol..

[53] Junqueira J.C., Fuchs B.B., Muhammed M., Coleman J.J., Suleiman J.M., Vilela S.F., Costa A.C., Rasteiro V.M., Jorge A.O., Mylonakis E. (2011). Oral Candida Albicans Isolates from HIV-Positive Individuals Have Similar in Vitro Biofilm-Forming Ability and Pathogenicity as Invasive Candida Isolates. BMC Microbiol..

[54] Marcos-Zambrano L.J., Bordallo-Cardona M.Á., Borghi E., Falleni M., Tosi D., Muñoz P., Escribano P., Guinea J. (2020). Candida Isolates Causing Candidemia Show Different Degrees of Virulence in Galleria Mellonella. Med. Mycol..

[55] Perini H.F., Moralez A.T.P., Almeida R.S.C., Panagio L.A., Junior A.O.G., Barcellos F.G., Furlaneto-Maia L., Furlaneto M.C. (2019). Phenotypic Switching in Candida Tropicalis Alters Host-Pathogen Interactions in a Galleria Mellonella Infection Model. Sci. Rep..

[56] Németh T., Tóth A., Szenzenstein J., Horváth P., Nosanchuk J.D., Grózer Z., Tóth R., Papp C., Hamari Z., Vágvölgyi C. (2013). Characterization of Virulence Properties in the C. Parapsilosis Sensu Lato Species. PLoS ONE.

[57] Cirasola D., Sciota R., Vizzini L., Ricucci V., Morace G., Borghi E. (2013). Experimental Biofilm-Related Candida Infections. Future Microbiol..

[58] Rossoni R.D., Barbosa J.O., Vilela S.F.G., dos Santos J.D., Jorge A.O.C., Junqueira J.C. (2013). Correlation of Phospholipase and Proteinase Production of Candida with in Vivo Pathogenicity in Galleria Mellonella. Braz. J. Oral Sci..

[59] Mroczyńska M., Brillowska-Dabrowska A. (2019). First Report on Echinocandin Resistant Polish Candida Isolates. Acta Biochim. Pol..

[60] Price M.F., Wilkinson I.D., Gentry L.O. (1982). Plate Method for Detection of Phospholipase Activity in Candida Albicans. Sabouraudia J. Med Vet. Mycol..

[61] Pham L.T.T., Pharkjaksu S., Chongtrakool P., Suwannakarn K., Ngamskulrungroj P. (2019). A Predominance of Clade 17 Candida Albicans Isolated From Hemocultures in a Tertiary Care Hospital in Thailand. Front. Microbiol..

[62] De Melo Riceto É.B., de Paula Menezes R., Penatti M.P.A., dos Santos Pedroso R. (2015). Enzymatic and Hemolytic Activity in Different Candida Species. Rev. Iberoam. Micol..

[63] Ranjith K., Chakravarthy S.K., Adicherla H., Sharma S., Shivaji S. (2018). Temporal Expression of Genes in Biofilm-Forming Ocular Candida Albicans Isolated from Patients with Keratitis and Orbital Cellulitis. Invest. Ophthalmol. Vis. Sci..

