# Supplementary material for: Virulence of Clinical Candida Isolates
Source: Pathogens. 2021 Apr 12;10(4):466. doi: 10.3390/pathogens10040466 (PMC8070227; doi:10.3390/pathogens10040466)
Supplement: Supplementary file 1 [file pathogens-10-00466-s001.zip › Supplementary Materials S1.docx]

**Table S1.** The survival of larvae infected with individual *Candida* isolates after the 2nd, 4th and 6th day of infections and the standard deviation from two independent experiment.

| MIC range | Species | Isolate No. | After 2^nd^ | SD | After 4^th^ | SD | After 6^th^ | SD |
| --- | --- | --- | --- | --- | --- | --- | --- | --- |
| MIC value ≤0.016 mg/l | *C. albicans* | 71 | 1.0 | - | 0.7 | 0.1 | 0.6 | 0.1 |
|  | *C. albicans* | 380 | 1.0 | - | 0.9 | 0.067 | 0.1 | 0.067 |
|  | *C. albicans* | 389 | 1.0 | - | 0.85 | 0.08 | 0.54 | 0.114 |
|  | *C. albicans* | 1010 | 1.0 | - | 0.75 | - | 0.50 | 0.5 |
|  | *C. albicans* | 1296 | 1.0 | - | 0.74 | 0.1 | 0.26 | 0.1 |
|  | *C. albicans* | 1768 | 1.0 | - | 0.85 | 0.08 | 0.40 | 0.1 |
|  | *C. albicans* | 2023 | 1.0 | - | 0.50 | 0.117 | 0.15 | 0.08 |
|  | *C. albicans* | 2029 | 1.0 | - | 0.74 | 0.11 | 0.32 | 0.107 |
|  | *C. albicans* | 2608 | 1.0 | - | 0.60 | 0.11 | 0.15 | 0.084 |
|  | *C. palmioleophila* | 370 | 1.0 | - | 0.8 | 0.09 | 0.40 | 0.1 |
|  | *C. parapsilosis* | 395 | 1.0 | - | 1.0 | - | 1.0 | - |
| MIC value 0.31-0.25 mg/l | *C. albicans* | 40 | 0.95 | 0.049 | 0.6 | 0.11 | 0.16 | 0.086 |
|  | *C. albicans* | 49 | 0.8 | 0.09 | 0.50 | 0.11 | 0.40 | 0.11 |
|  | *C. albicans* | 114 | 0.4 | 0.11 | 0.0 | - | 0.0 | - |
|  | *C. albicans* | 125 | 1.0 | - | 0.39 | 0.115 | 0.056 | 0.054 |
|  | *C. albicans* | 286 | 1.0 | - | 0.95 | 0.051 | 0.33 | 0.1 |
|  | *C. glabrata* | 1150 | 1.0 | - | 0.58 | 0.11 | 0.21 | 0.094 |
| MIC value ≥ 0.5 mg/l | *C. albicans* | 54 | 0.45 | 0.11 | 0.1 | 0.067 | 0.0 | 0.00 |
|  | *C. krusei* | 102 | 1.0 | - | 1.0 | - | 1.0 | - |
|  | *C. palmioleophila* | 4 | 1.0 | - | 0.8 | 0.089 | 0.44 | 0.113 |
|  | *C. palmioleophila* | 368 | 0.95 | 0.049 | 0.90 | 0.067 | 0.90 | 0.067 |
|  | *C. parapsilosis* | 105 | 1.0 | - | 1.0 | - | 1.0 | - |
|  | *C. parapsilosis* | 443 | 1.0 | - | 0.9 | 0.067 | 0.7 | 0.102 |
|  | *C. parapsilosis* | 441 | 1.0 | - | 0.65 | 0.107 | 0.65 | 0.107 |
|  | *C. inconspicua* | 1444 | 0.95 | 0.049 | 0.40 | 0.08 | 0.40 | 0.110 |
| Control | *Untouched larvae* |  | 1.0 | - | 1.0 | - | 1.0 | - |
|  | *PBS* |  | 1.0 | - | 1.0 | - | 1.0 | - |
|  | *Pierced larvae* |  | 1.0 | - | 1.0 | - | 1.0 | - |

SD – standard deviation, After 2^nd^ – survival after second day of larvae infection by tested isolates

The survival equal to 1 is marked in yellow. The survival ≥0.7 to 0.95 (low virulence) is marked with green, light red marks a survival rate between 0.4-0.7 (moderately virulent), and dark red marks a survival of less than 0.4 (highly virulent).
