# Supplementary material for: Virulence of Clinical Candida Isolates
Source: Pathogens. 2021 Apr 12;10(4):466. doi: 10.3390/pathogens10040466 (PMC8070227; doi:10.3390/pathogens10040466)
Supplement: Supplementary file 1 [file pathogens-10-00466-s001.zip › Supplementary Materials S2.docx]

**Table S2.** The Person`s correlation and P value between tested variabilities

| Variables | | CSP | MCF | Hz | Pz | PRz | Ez | Biofilm | Survival |
| --- | --- | --- | --- | --- | --- | --- | --- | --- | --- |
| AND | correlation | 0.931 | 0.998 | 0.203 | 0.151 | 0.314 | 0.403 | 0.502 |  |
|  | P value | **0.0001** | **0.0001** | 0.342 | 0.501 | 0.135 | 0.0564 | **0.0125** |  |
| CSP | correlation |  | 0.932 | 0.330 | 0.192 | 0.251 | 0.489 | 0.422 |  |
|  | P value |  | **0.0001** | 0.115 | 0.392 | 0.237 | **0.0180** | **0.0399** |  |
| MCF | correlation |  |  | 0.227 | 0.157 | 0.334 | 0.423 | 0.504 |  |
|  | P value |  |  | 0.287 | 0.485 | 0.111 | **0.0443** | **0.0121** |  |
| Hz | correlation |  |  |  | -0.177 | 0.430 | 0.330 | -0.0876 | 0.474 |
|  | P value |  |  |  | 0.431 | 0.0357 | 0.124 | 0.684 | **0.0193** |
| Pz | correlation |  |  |  |  | 0.129 | 0.242 | 0.0804 | 0.245 |
|  | P value |  |  |  |  | 0.566 | 0.291 | 0.722 | 0.272 |
| Prz | correlation |  |  |  |  |  | 0.422 | 0.253 | 0.505 |
|  | P value |  |  |  |  |  | **0.0446** | 0.233 | **0.0119** |
| Ez | correlation |  |  |  |  |  |  | 0.335 | 0.621 |
|  | P value |  |  |  |  |  |  | 0.118 | **0.00157** |
| Biofilm | correlation |  |  |  |  |  |  |  | 0.193 |
|  | P value |  |  |  |  |  |  |  | 0.366 |

AND - anidulafungin; CSP - caspofungin; MCF - micafungin; Hz - haemolytic activity, PZ - phospholipases activity; Prz - proteinase activity, Ez - esterase activity. The red colour means the positive
correlation between two variabilities and also P value <0.05.
