# Supplementary material for: Virulence of Clinical Candida Isolates
Source: Pathogens. 2021 Apr 12;10(4):466. doi: 10.3390/pathogens10040466 (PMC8070227; doi:10.3390/pathogens10040466)
Supplement: Supplementary file 1 [file pathogens-10-00466-s001.zip › Supplementary Materials S3.docx]

**Table S3.** Enzymatic activity from *Candida* isolates and the standard deviation (SD) from independent experiments.

| *Candida spp.* | Isolate No**.** | **Haemolytic activity** | | **Phospholipase activity** | | **Protease activity** | | **Esterase activity** | | **Biofilm production** | |
| --- | --- | --- | --- | --- | --- | --- | --- | --- | --- | --- | --- |
|  |  | H_z_ value | SD | P_z_ value | SD | PR_z_  value | SD | E_z_ value | SD | Abs_600_ | SD |
| *C. albicans* | **40** | 0.44 | 0.03 | 1 | 0 | 0.37 | 0.02 | 0.53 | 0.04 | 0.97 | 0.25 |
| *C. albicans* | **49** | 0.56 | 0 | 1 | 0 | 0.34 | 0.05 | 0.48 | 0.03 | 0.24 | 0.12 |
| *C. albicans* | **54** | 0.48 | 0.03 | 1 | 0 | 0.32 | 0.01 | weak growth | | 1.56 | 0.92 |
| *C. albicans* | **71** | 0.59 | 0.05 | 1 | 0 | 0.35 | 0.02 | 0.40 | 0.04 | 0.51 | 0.08 |
| *C. albicans* | **114** | 0.67 | 0.06 | lack of growth | | 0.45 | 0 | 0.53 | 0.04 | 0.12 | 0.09 |
| *C. albicans* | **125** | 0.61 | 0.04 | 0.81 | 0.27 | 0.36 | 0.02 | 0.39 | 0.04 | 1.05 | 0.04 |
| *C. albicans* | **286** | 0.48 | 0.03 | 1 | 0 | 0.35 | 0.02 | 0.44 | 0.03 | 0.97 | 0.20 |
| *C. albicans* | **380** | 0.52 | 0.03 | 1 | 0 | 0.39 | 0.02 | 0.45 | 0.02 | 0.89 | 0.36 |
| *C. albicans* | **389** | 0.48 | 0.03 | 1 | 0 | 0.36 | 0.02 | 1 | 0 | 1.89 | 0.33 |
| *C. albicans* | **1010** | 0.48 | 0.03 | 0.48 | 0.03 | 0.39 | 0.02 | 0.44 | 0.03 | 1.27 | 0.58 |
| *C. albicans* | **1296** | 0.44 | 0.03 | 0.80 | 0.08 | 0.48 | 0.03 | 0.55 | 0 | 0.80 | 0.05 |
| *C. albicans* | **1768** | lack of growth | | lack of growth | | lack of growth | | lack of growth | | 0.11 | 0.33 |
| *C. albicans* | **2023** | 1 | 0 | 0.48 | 0.03 | 0.36 | 0.02 | 0.44 | 0.03 | 0.49 | 0.18 |
| *C. albicans* | **2029** | 0.57 | 0.04 | 0.88 | 0.18 | 0.35 | 0 | 0.37 | 0.02 | 1.34 | 0.37 |
| *C. albicans* | **2608** | 0.48 | 0.03 | 1 | 0 | 0.38 | 0 | 0.46 | 0.06 | 1.04 | 0.44 |
| *C. glabrata* | **1150** | 0.50 | 0 | lack of growth | | 0.35 | 0 | 1 | 0 | 0.81 | 0.06 |
| *C. krusei* | **102** | 0.52 | 0.03 | 1 | 0 | 0.40 | 0 | 1 | 0 | 0.98 | 0.09 |
| *C. palmioleop.* | **4** | 1 | 0 | 0.63 | 0.05 | 0.40 | 0.02 | 0.52 | 0.04 | 1.05 | 0.04 |
| *C. palmioleop.* | **368** | 1 | 0 | 1 | 0 | 0.48 | 0.03 | 1 | 0 | 0.97 | 0.35 |
| *C. palmioleop.* | **370** | 0.67 | 0.06 | 0.51 | 0.07 | 0.37 | 0.02 | 1 | 0 | 1.15 | 0.62 |
| *C. parapsilosis* | **105** | 1 | 0 | 1 | 0 | 0.59 | 0.03 | 1 | 0 | 2.33 | 0.29 |
| *C. parapsilosis* | **395** | 1 | 0 | 1 | 0 | 0.50 | 0 | 1 | 0 | 0.42 | 0.20 |
| *C. parapsilosis* | **441** | 0.59 | 0.05 | 1 | 0 | 0.39 | 0.04 | 0.63 | 0.05 | 1.01 | 0.05 |
| *C. parapsilosis* | **443** | 1 | 0 | 1 | 0 | 0.35 | 0.03 | 1 | 0 | 0.69 | 0.06 |
| *C. inconspicua* | **1444** | 0.56 | 0 | 1 | 0 | 0.48 | 0.03 | 1 | 0 | 1.58 | 0.70 |

*C. palmioleop*.- *C. palmioleophila*; Hz- index of haemolytic activity; Pz- index of phospholipase activity; PRz – index of protease activity; Ez – index of esterase; Dark red marks a strong activity (≤0.63), light red marks a weak activity (0.64 <value <0.99) and green marks a non- activity (value = 1). Orange marks a strong biofilm production (OD value ≥ 1.14), yellow weak biofilm production (0.76> OD value <1.14) and blue marks negative biofilm production (OD value ≤0.76). Dark red marks a survival of less than 0.4 (highly virulent), light red marks a survival rate between 0.4-0.7 (moderately virulent). The survival ≥0.7 to 0.95 (low virulence) is marked with green. The survival equal to 1 is marked in yellow.
