# Supplementary material for: Virulence of Clinical Candida Isolates
Source: Pathogens. 2021 Apr 12;10(4):466. doi: 10.3390/pathogens10040466 (PMC8070227; doi:10.3390/pathogens10040466)
Supplement: Supplementary file 1 [file pathogens-10-00466-s001.zip › Supplementary Materials S4.docx]

**Table S4**. Exemplary pictures of the virulence factor study on various substrates.

|  | **Investigation of virulence factors producing by 25 *Candida* isolates.** | | |
| --- | --- | --- | --- |
| Description of individual isolates on different medium. | 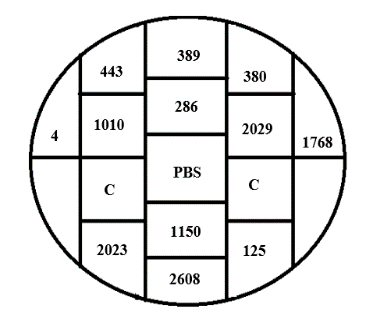PBS – the sterility control of phosphate-buffered saline  C – control of medium | 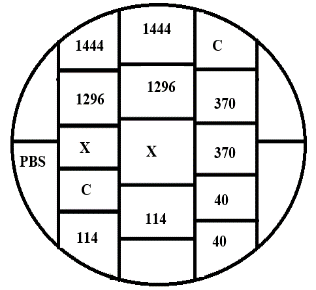X - another isolates using as control | 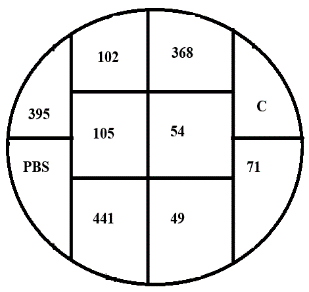 |
| The photo of *Candida* growth on the sheep blood medium. | 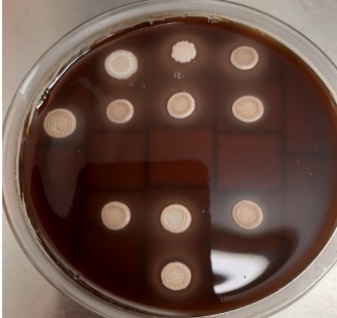 | 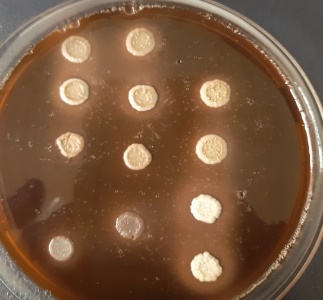 | 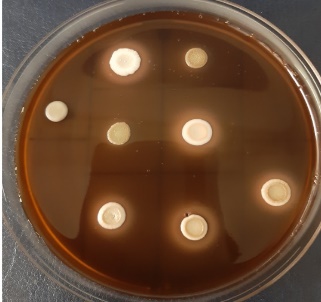 |
| The photo of *Candida* growth on the egg yolk medium. | 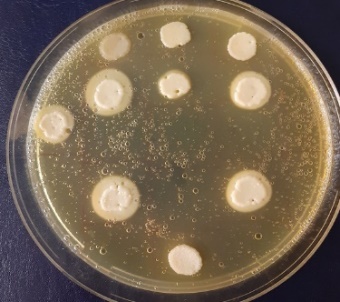 | 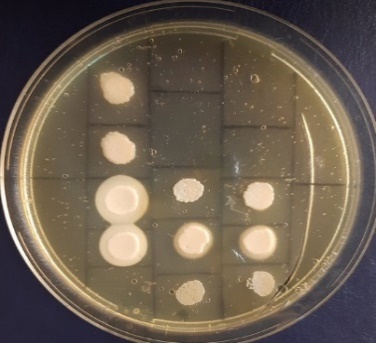 | 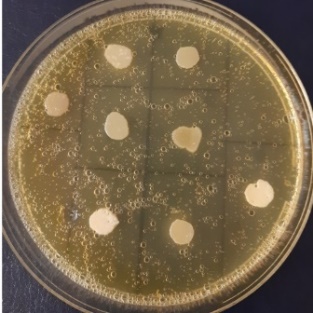 |

continuation of the table

| The photo of *Candida* growth on the BSA medium.* | 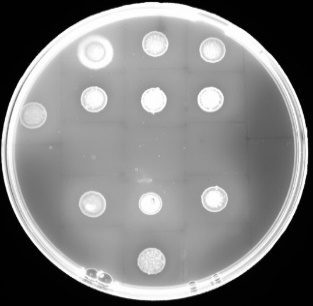 | 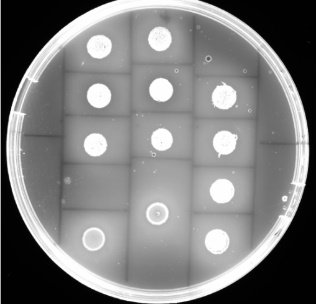 | 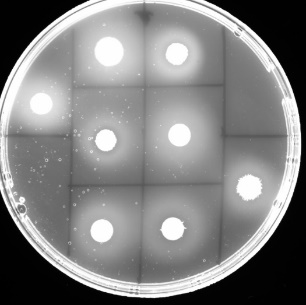 |
| --- | --- | --- | --- |
|  | 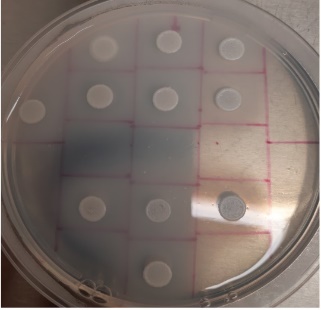 | 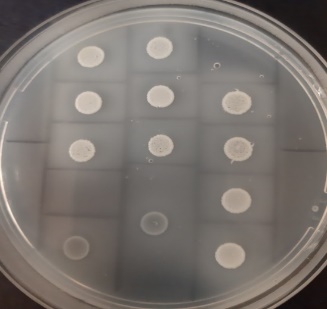 | 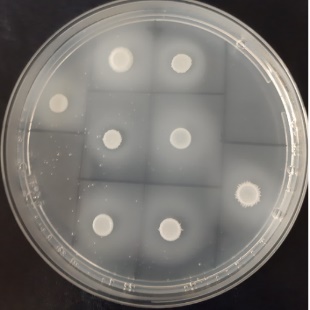 |
| The photo of *Candida* growth on the tween 80 medium.* | 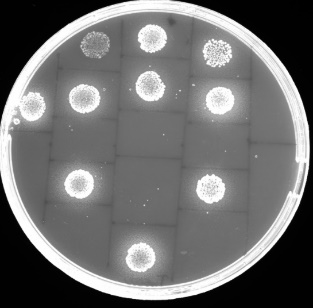 | 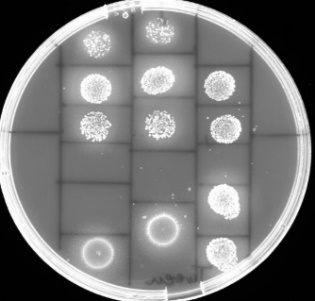 | 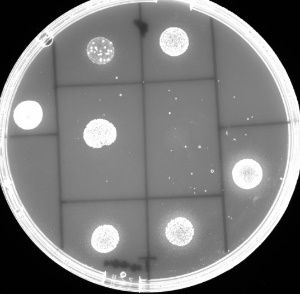 |
|  | 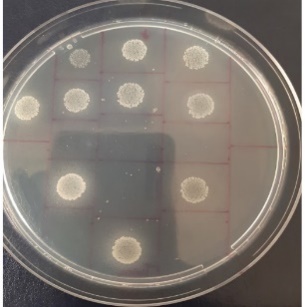 | 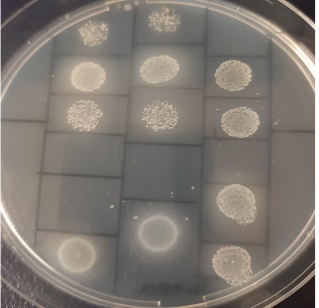 | 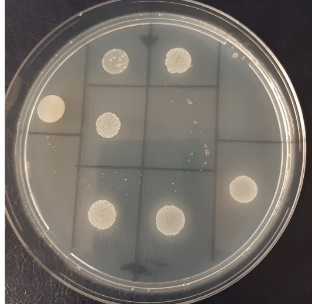 |

*The photos were made by two different way: the photo above by ChemiDOC and photo below by camera.
